# Strengthening Amazon conservation through community‐based voluntary patrolling

**DOI:** 10.1111/cobi.70045

**Published:** 2025-05-30

**Authors:** Caetano L. B. Franco, Thais Q. Morcatty, Helder L. Queiroz, Michael G. Sorice, Julia E. Fa, Paulo Roberto e Souza, Isabel S. Sousa, João Valsecchi, Hani R. El Bizri

**Affiliations:** ^1^ Instituto de Desenvolvimento Sustentável Mamirauá Tefé Brazil; ^2^ Department of Forest Resources and Environmental Conservation Virginia Tech Blacksburg Virginia USA; ^3^ Department of Geography University College London London UK; ^4^ Research Network on Diversity, Conservation and Use of Amazonian Wildlife Manaus Brazil; ^5^ Center for International Forestry Research Bogor Barat Indonesia; ^6^ School of Science and the Environment Manchester Metropolitan University Manchester UK; ^7^ Natural Sciences and Environment Research Hub University of Gibraltar Gibraltar

**Keywords:** Amazonia, community‐based management, enforcement, environmental crime, local communities, patrolling, protected area, wildlife crime, Amazonia, aplicación de la ley, área protegida, comunidades locales, gestión comunitaria, vigilancia, 亚马逊, 基于社区的管理, 巡逻, 执法, 当地社区, 保护地

## Abstract

Globally, environmental crimes are a major threat to biodiversity and the livelihood of local populations. Community‐based protection of natural resources, which involves local people in surveillance and enforcement, is an important complement to the government‐led command‐and‐control policing approach. We examined whether a community‐based voluntary patrolling system deterred environmental crimes in Amazonia. We used data on environmental crimes recorded by patrollers over 11 years (2003–2013) in 12 independent territorial units in 2 large protected areas (PAs) in Amazonas, Brazil. For comparison, we also analyzed data from government‐led enforcement operations outside these PAs from 2002 to 2012. In total, patrollers conducted almost 20,000 surveillance outings (around 150,000 h of activity) and recorded the occurrence of 1260 crimes. Of the 772 crimes for which we had data on seized items, most violations were related to fishing (78.24%), 19.04% to hunting, or 2.72% to logging. The occurrence of crimes per outing increased as the number of patrollers and time spent patrolling increased and was greater during outings that were informant led. There was a sharp decrease over time in the occurrence of crimes during patrols across 11 of the 12 territorial units examined. Overall, the occurrence of crimes declined by approximately 80% over the study period. In contrast, the number of crimes detected over time during government‐led enforcement operations outside the PAs did not decline. Leadership of local communities in the planning and conducting of patrols contributed to rule conformity and enforcement in the PAs. Our results should be especially useful to managers of PAs and researchers in other parts of the tropics as a model for local patrolling and natural resource protection.

## Abstract (es)

Fortalecimiento de la conservación en la Amazonia por medio de la vigilancia comunitaria voluntaria

**Resumen:**

En todo el mundo, los delitos contra el medio ambiente constituyen una grave amenaza para la biodiversidad y los medios de subsistencia de las poblaciones locales. La protección comunitaria de los recursos naturales, que involucra a la población local en la vigilancia y la aplicación de la ley, es un complemento importante del enfoque policial de mando y control dirigido por el gobierno. Nuestro estudio analiza si un sistema de vigilancia comunitaria voluntaria disuade la comisión de delitos contra el medio ambiente en la Amazonia. Utilizamos datos sobre delitos ambientales registrados por vigilantes durante 11 años (2003‐2013) en 12 unidades territoriales independientes en dos grandes áreas protegidas en Amazonas, Brasil. A modo de comparación, también analizamos datos de operaciones de aplicación de la ley dirigidas por el gobierno fuera de estas áreas protegidas entre 2002 y 2012. En total, los vigilantes realizaron casi 20,000 salidas de vigilancia (unas 150,000 horas de actividad) y registraron la comisión de 1,260 delitos. De los 772 delitos de los que disponíamos de datos sobre objetos incautados, la mayoría de las infracciones estaban relacionadas con la pesca (78.24%), el 19.04% con la caza y el 2.72% con la tala. El número de delitos por salida aumentó a medida que aumentaba el número de vigilantes y el tiempo dedicado a patrullar, y fue mayor durante las salidas dirigidas por informantes. En 11 de las 12 unidades territoriales examinadas se produjo un fuerte descenso de los delitos durante los patrullajes. En conjunto, la incidencia de delitos disminuyó aproximadamente un 80% durante el periodo de estudio. En cambio, el número de delitos detectados a lo largo del tiempo durante las operaciones de aplicación de la ley dirigidas por el gobierno fuera de las áreas protegidas no disminuyó. El liderazgo de las comunidades locales en la planificación y realización de patrullajes contribuyó a la conformidad con las normas y a su cumplimiento en las áreas protegidas. Nuestros resultados deberían ser especialmente útiles para los gestores de áreas protegidas y los investigadores de otras zonas tropicales como modelo para la vigilancia local y la protección de los recursos naturales.

## 通过基于社区的自愿巡逻加强亚马逊地区的保护

**【摘要】** 在全球范围内, 环境犯罪是对生物多样性和当地居民生计的一项重大威胁。基于社区的自然资源保护, 即让当地居民参与监督和执法, 是对政府主导的指挥和控制管辖方法的重要补充。本研究分析了一个基于社区的自愿巡逻系统是否能阻止亚马逊地区的环境犯罪。研究使用了巴西亚马逊地区两个大型保护地12个独立区域单元的巡逻人员在11年间(2003–2013年)记录的环境犯罪数据。为进行比较, 我们还分析了2002–2012年间在这些保护地之外由政府主导的执法行动的数据。巡逻人员共进行了近20,000次监督巡视(约150,000小时), 记录了1,260起犯罪事件。在记录了缴获物品数据的772起犯罪事件中, 大多数违法行为与捕鱼有关(占78.24%), 还有19.04%与狩猎有关, 2.72%与伐木有关。随着巡逻人员数量和巡逻时间增加, 每次巡逻活动记录的犯罪事件数量也在增加, 且在知情者领导的巡逻中发现的犯罪事件更多。在我们分析的12个区域单元中, 有11个单元巡逻期间的犯罪事件随时间推移而急剧减少。总体而言, 我们研究期间的犯罪事件减少了约80%。相比之下, 在政府主导的保护地之外的执法行动中发现的犯罪数量并没有随时间推移而减少。我们提出, 当地社区在巡逻计划和执行中的领导作用有助于落实保护地内规则和法律的执行。我们的研究结果可以作为当地巡逻和自然资源保护的典范, 对保护地管理者和其他热带地区的研究人员有很大帮助。**【翻译:胡怡思;审校:聂永刚】**

## INTRODUCTION

Globally, noncompliance with government or local regulations and norms and laws concerning the protection of nature is one of the main threats to biodiversity (Esmail et al., 2020; Gavin et al., 2010). Environmental crimes are usually offenses against property rights and include the harvest of natural resources in prohibited places or times or their harvest with unauthorized equipment or methods (Gavin et al., 2010). Illegal use also includes harvesting of legally protected species (Gavin et al., 2010). The impact of such crimes is diverse, affecting the status of species populations (Dinerstein et al., 2007; El Bizri et al., 2024), management of protected areas (PAs) (Bergseth et al., 2018; Critchlow et al., 2017; Kauano et al., 2017; Yonariza & Webb, 2007), and provision of ecosystem services. Environmental crimes also have social and economic implications for civil society, governments, and international organizations (Gore et al., 2019). They can exhaust resources vital for the survival of communities; impair the ability of governments and nongovernmental organizations to function effectively (Nellemann et al., 2016); and foster resentment and resistance in communities, which can undermine long‐term cooperation and compliance with environmental initiatives. Effective public policies and efficient networks of governmental and nongovernmental actors are required to guard, detect, and deter violators. In most tropical nations, environmental enforcement is often hampered by high levels of corruption and a lack of appropriate environmental legislation (Abessa et al., 2019; Morcatty et al., 2020; Vale et al., 2021).

The challenge of conserving biodiversity in PAs involves enforcing regulations against environmental crimes (Kauano et al., 2017; Pulido‐Chadid et al., 2023), but the root causes of these activities, such as poverty, food insecurity, and cultural degradation, cannot be overlooked. Effective conservation strategies must tackle underlying socioeconomic and cultural factors to reduce illegal wildlife use and other environmental crimes. By addressing root causes, these strategies not only mitigate immediate threats to biodiversity but also foster sustainable community development, leading to more enduring conservation outcomes (Duffy et al., 2016). This task is complicated by difficulties in imposing penalties and detecting activities, especially covert activities, such as logging and hunting. Many tropical PAs face enforcement limitations due to budget constraints, and implementing regulations can lead to conflicts, putting staff at risk and resulting in a reluctance to enforce rules in high‐conflict areas (Jiao et al., 2021; Tranquilli et al., 2014). These challenges are compounded by the major issue of poor or nonexistent management plans, which often result in so‐called paper parks (Dudley & Stolton, 1999; Watson et al., 2014).

The relationship between compliance and legitimacy in PAs is complex and multifaceted. Conventional government‐led enforcement methods (i.e., command and control) alone may not be sufficient to ensure compliance with PA regulations. Instead, the trustworthiness of PA managers is a significant predictor of voluntary compliance (Cinner et al., 2012). This trust is often based on the public's perceptions of positive interactions with PA managers, their receptiveness to local input, the benefits and disadvantages of the PA's presence, and the equitable treatment of different groups. Trust and legitimacy are key because when people believe a system or rule is fair, they are more likely to follow it willingly (e.g., Stern, 2008).

The Amazon is the largest tropical forest in the world. The region is responsible for the provision of ecosystem services of global importance (Ferrante & Fearnside, 2019; Levis et al., 2020), including 20% of the world's freshwater fish stocks (Charity et al., 2016) and over 10% of vertebrates and plants (Guayasamin et al., 2021). It also serves as a crucial carbon sink, sequestering vast amounts of carbon in its biomass and moderating global climate (Gatti et al., 2021; Moraes et al., 2021). Furthermore, the more than 34 million people living in rural areas or Indigenous lands in the Amazon depend on the region's natural resources for their food and livelihoods (Charity et al., 2016). In the Brazilian Amazon, environmental crimes are typically addressed through command‐and‐control measures carried out by governmental environmental agencies and law enforcement authorities. Nevertheless, given that roughly 60% of the Amazon is in Brazil and that the area is vast and difficult to access, ensuring effective protection of this territory remains a significant challenge (da Silva & Bernard, 2016; Kauano et al., 2017; Regueira & Bernard, 2012; WWF, 2009).

The number of official enforcement agents in the Brazilian Amazon is one environmental agent (inspector) per 2100 km^2^ and one environmental military police officer per 4097 km^2^. These figures are more than 2000% lower than those recommended by international environmental protection agencies, such as the International Union for Conservation of Nature (IUCN) (∼1 environmental officer/10–30 km^2^) and the International Ranger Federation (1 park ranger/100 km^2^) (de Oliveira et al., 2021). Only 10% of all fines imposed by the federal environmental enforcement agency (IBAMA) have been paid by violators (Barreto et al., 2009). Together, these factors generate a defective surveillance situation that ultimately compromises governance over the Amazon territory and its natural resources.

Almost 30 types of illegal use of natural resources have been identified in PAs in the Brazilian Amazon: 37.3% related to forest degradation, 27.3% to fishing, and 18.1% to hunting (Kauano et al., 2017). Illegal use also occurs outside PAs. For instance, almost 80% of *pirarucu* (giant arapaima [*Arapaima gigas*], the world's largest scaled freshwater fish) landed in the lower Amazon River is illegally caught (Cavole et al., 2015). Even though the sale of wild meat has been prohibited in Brazil for over 60 years, more than 8000 t are traded annually in central Amazonian cities for consumption by urban people, generating a yearly revenue exceeding US$30 million (Brazil, 1967; El Bizri et al., 2020).

Decentralization of conservation efforts and greater social participation can effectively protect tropical forests (Agrawal & Ribot, 1999; Larson & Soto, 2008). The involvement of local populations can ensure compliance with natural resource management and prevent environmental crimes by incorporating local knowledge and practices, fostering a sense of ownership, and building trust between conservation authorities and local communities (Bergseth et al., 2018), particularly in localities where official government‐led enforcement is absent or ineffective (Cinner et al., 2012; Norris et al., 2018). Local management plans and rules are usually more closely adhered to than national ones, further reinforcing the effectiveness of community‐based approaches. Furthermore, when community enforcement works together with government rules, it can be more effective because it matches what the community believes is right and important to be protected (see Bergseth et al., 2018; Nolte, 2016; Stern, 2008).

Involvement in surveillance and environmental law enforcement often poses serious implications for community agents (Bergseth et al., 2018) because they are subject to great risks while conducting their activities (Masse et al., 2017). Official environmental protection agents are also threatened. For example, rangers (professionals engaged in the protection and management of protected and conserved areas) play an indispensable role in conservation in many parts of the world (Singh et al., 2020); however, their safety is neglected almost everywhere (Digun‐Aweto et al., 2019). These rangers face excessive insecurity, threats to their lives, and health risks (Belecky et al., 2019). Therefore, integrating ranger patrols with the community expectations can help build trust between authorities and local communities, which facilitates information sharing, reduces conflict or violent reprisals, and ensures that sanctions are fair and prompt (Moreto et al., 2016).

In the Brazilian Amazon, local people have been engaged in formal environmental protection actions in their territories for at least 40 years (Franco et al., 2021). These actions were legally formalized through the Voluntary Environmental Agents (VEA) Program, a community‐based protection system endorsed by the Brazilian government (Amazonas, 2007, 2008; Brazil, 2001, 2005). In this program, local people are involved in territorial monitoring of environmental crimes in PAs or in areas where community‐based management programs of natural resources, such as fishing agreements, operate. Evidence supports the effectiveness of these community‐based management programs in the Amazon. For instance, studies by Campos‐Silva and Peres (2016) and Campos‐Silva et al. (2021) show that community‐based management induces the rapid recovery of high‐value tropical freshwater fisheries (demonstrating the effectiveness of locally driven conservation efforts in revitalizing vital ecological and economic resources) and improves the sustainability of local livelihoods. These findings align with the broader literature on the benefits of decentralization and community participation in conservation efforts (Agrawal & Gibson, 1999; Hayes & Ostrom, 2005). However, the effectiveness of community‐based voluntary patrolling, such as the VEA Program, in reducing environmental crimes within these frameworks remains uncertain.

We investigated whether community‐based voluntary patrolling by local people in the Brazilian Amazon effectively curbs environmental crimes. Community agents in law enforcement typically focus on local monitoring and education, whereas government agents often have broader legal authority and resources for enforcement actions. We used VEA Program data on the occurrence of environmental crimes over 11 years in 2 large PAs and data collected on the crimes detected by the official governmental surveillance system of the Brazilian environmental agency for comparison (hereafter government‐led enforcement operations). We consider an environmental crime any activity that breaches communal, regional, or governmental rules and laws about harming the environment. We believe our assessment can provide a model for community‐based patrolling for natural resource protection for conservation practitioners, PA managers, and researchers in other parts of the tropics.

## METHODS

### Study area

**FIGURE 1:**
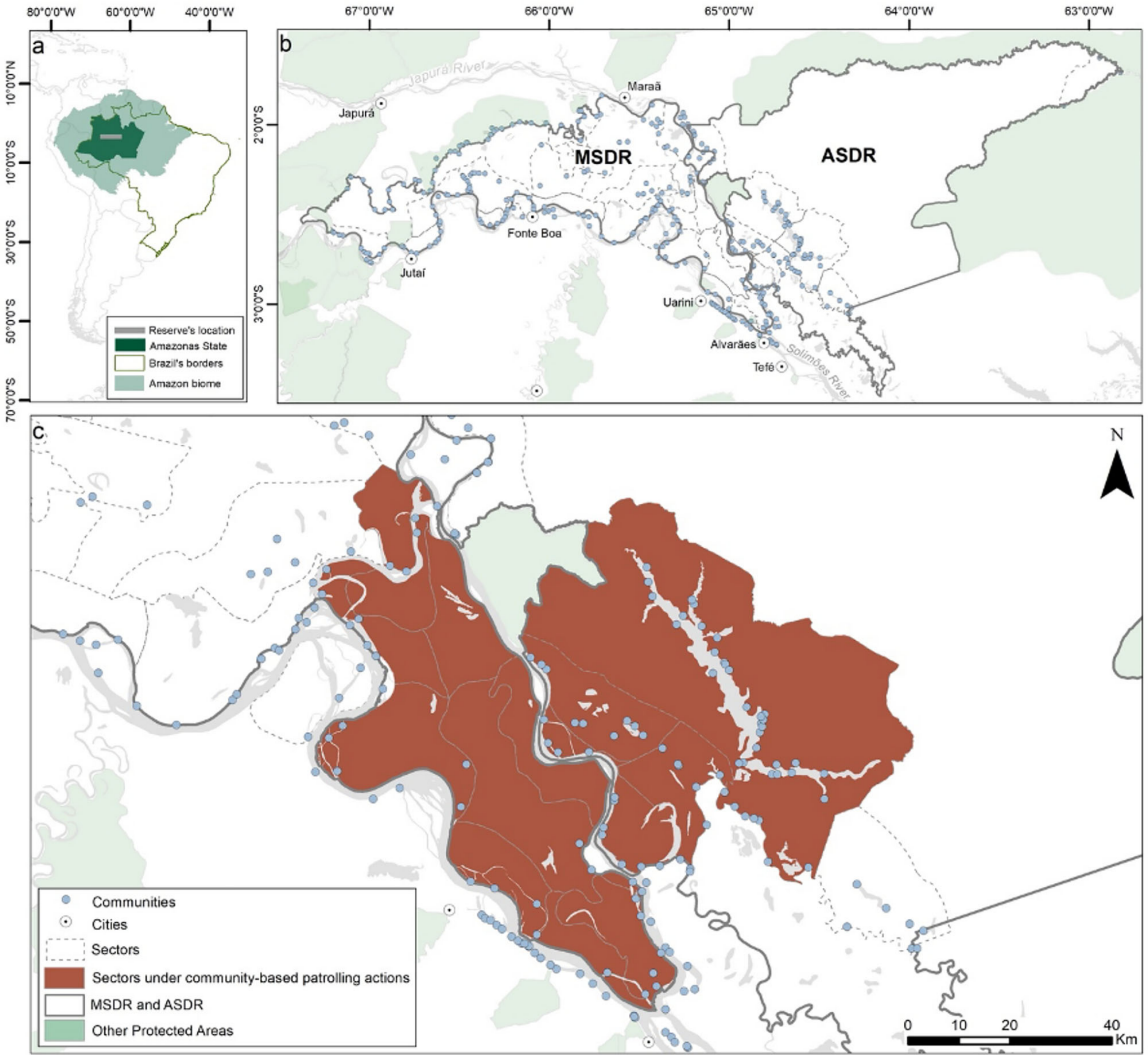
The location of the (a) Amazon biome and Amazonas state, (b) Mamirauá Sustainable Development Reserve (MSDR) and Amanã Sustainable Development Reserve (ASDR), and (c) area in these reserves under voluntary patrolling by people in local communities.

This study took place in 2 adjacent sustainable development reserves (category VI, IUCN) in the state of Amazonas in the Brazilian Amazon. The Mamirauá Sustainable Development Reserve (MSDR) (03°08ʹS, 64°45ʹW) is 11,240 km^2^ of white‐water flooded forest with a population of 11,304 spread over 211 communities and settlements (Figure 1b) (MISD, 2019). The Amanã Sustainable Development Reserve (ASDR) (02°21ʹS, 64°16ʹW) covers 23,500 km^2^ and includes flooded and nonflooded ecosystems (Figure 1b). The ASDR has 5458 inhabitants in 133 communities and settlements (MISD, 2018). These communities have lived in the region for a long time, mostly occupying the banks of rivers, and are known locally as *ribeirinhos*. The territorial management of these 2 areas was guided by the local communities and their history of use and occupation of the territory (Queiroz, 2005).

The 2 PAs are divided into territorial governing units that aggregate adjacent communities into geopolitical organizations that, for the most part, predate the creation of these PAs (Figure 1c). These traditional territories are officially recognized and legitimized through the implementation of PAs. The people living in the territorial units supervise patrols therein and participate in the decision‐making and management processes of the PAs. Fishing, hunting, farming, and logging are economic and subsistence activities carried out by residents in these reserves (Alencar, 2010; Queiroz & Peralta, 2006). In the immediate surroundings of the PAs are 5 small towns: Alvarães (16,220 inhabitants), Fonte Boa (17,005), Jutaí (13,886), Maraã (18,261), and Uarini (13,690). The city of Tefé (59,547 inhabitants), which is approximately 50 km away from these 2 reserves, is an important regional hub for the flow of rural production and the provision of urban services.

### VEA Program

The VEA Program was established in the 2 PAs in 1995. It is complementary to the existing governmental surveillance system (Franco et al., 2021; Souza & Queiroz, 2008) and is carried out by community residents and users (i.e., people living outside the limits of the PAs who, due to historical use in areas in the PAs, have the right to use natural resources in certain areas) (Amazonas, 2007, 2008; Brazil, 2001, 2005). The official environmental agencies at the federal and state levels are responsible for training agents and supporting their activities. The Mamirauá Institute for Sustainable Development (MISD), acting as a facilitating body, maintains a partnership with local communities and cooperates with the government to secure financial resources for patrolling activities, fostering social organization, and managing surveillance data (Franco et al., 2021).

The VEA Program is an innovative approach to conservation that leverages the diverse capabilities and roles of Indigenous people and local communities in managing PAs. Central to the program's objectives are patrolling, environmental education, social mobilization, leadership development, and conflict mediation. These elements are critical for fostering understanding of and engaging with local communities in conservation efforts. Through environmental education, the program empowers communities with knowledge about sustainable practices and the ecological importance of their actions. Social mobilization efforts enhance collective action by building a community network that supports conservation initiatives. Furthermore, the program prioritizes leadership development, which helps local leaders guide conservation efforts, mediate conflicts, and ensure that conservation strategies are culturally sensitive and legally sound. This comprehensive, community‐based approach addresses the immediate challenges of PA management and integrates the social dimensions necessary for sustainable conservation, making the VEA Program a robust inspirational model for community engagement in natural resources comanagement (Franco et al., 2021).

Each participating PA territorial unit has a team of VEAs from communities in and surrounding that territorial unit. The VEAs are primarily responsible for patrolling their territories, which involves routine monitoring and responding to reports from community members about illegal activities. Patrolling is a coordinated effort conducted by a designated group responsible for monitoring specific areas in their designated territorial unit. This activity is strategically carried out in locations identified as vulnerable due to, for example, high incidences of illegal activities or environmental sensitivity. The selection of patrol routes is also informed by informational cues, which may include recent reports of environmental violations or intelligence gathered from local sources. This targeted approach allows the patrol teams to manage resources and focus their efforts where they are most needed. After observing an environmental crime, the VEAs deter the crime by confiscating illegal products and materials, record the incident, and file a report that is forwarded to the governmental agency responsible for enforcement. The crimes detected by VEAs are related to the extraction of natural resources in prohibited locations, out of season, with below‐minimum‐size harvests (e.g., fishes), and the extraction of species prohibited by local agreements or legislation. In an area of ​​8879.5 km^2^, by 2020, the program had 215 active VEAs in the 2 PAs (26.5% women and 11.6% Indigenous) (Franco et al., 2021).

That only 26.5% of VEAs were women raises important considerations about gender representation. However, the number of female participants in the VEA Program has increased over time, reflecting progress toward greater inclusiveness and diversity. Franco et al. (2021) explored this issue in detail. Although the initial gender imbalance may have represented a limitation, the ongoing increase in female participation suggests the program is moving in the right direction. A more gender‐balanced participation can bring diverse perspectives to the monitoring of environmental crimes, potentially leading to more comprehensive and equitable outcomes. Although the program's gender representation and its implications are important considerations, we did not focus on gender aspects of the VEA Program. Our primary focus was evaluation of the overall effectiveness of VEA patrols in reducing environmental crimes. For a deeper exploration of the gender‐related aspects of the program, see Franco et al. (2021).

Individuals who engage in environmental crimes in this region can be members of the local communities, people from neighboring cities, or individuals from communities that do not reside in or use the PAs. There are communities that historically did not use natural resources in the areas now designated as PAs. Because these communities lacked a history of resource use in the PAs, they are not recognized as rightful users and, hence, are not allowed to extract resources under the management framework of the PAs.

### Data collection

We obtained data from participatory research led and collected by the MISD with the VEAs, who voluntarily filled in forms during their patrol activities from 2003 to 2013 in 12 territorial units in the 2 PAs (Table 1; Figure 1c). Each record referred to a patrol outing by a VEA group in their specific territorial unit. The information recorded included PA (MSDR or ASDR), territorial unit, date and time of departure from the community and time of return of the patrol, number of VEAs involved, reason for the outing (routine vs. informant led), whether a crime occurred, and the number of crimes.

**TABLE 1:** Description of the territorial units under community‐based voluntary patrolling for natural resource protection in Amanã Sustainable Development Reserve (ASDR) and Mamirauá Sustainable Development Reserve (MSDR).

Territorial unit	Period	Protected area	Area (km^2^)	Population (number of communities)^a^
Aranapú	2003–2013	MSDR	350.85	558 (8)
Barroso	2003–2013	MSDR	212.71	262 (7)
Boa União	2003–2013	MSDR	287.54	116 (2)
Horizonte	2003–2012	MSDR	424.23	482 (7)
Ingá	2003–2012	MSDR	131.65	774 (6)
Jarauá	2003–2013	MSDR	563.59	298 (5)
Liberdade	2003–2013	MSDR	223.22	1780 (17)
Mamirauá	2003–2011	MSDR	234.43	758 (12)
Tijuaca	2003–2013	ASDR; MSDR	482.61	577 (9)
Coraci	2003–2013	ASDR	321.08	413 (9)
Lago Amanã	2004–2013	ASDR	1210.74	748 (22)
São José	2004–2013	ASDR	256.87	652 (13)
Total	–	–	4699	7418 (117)

^a^ Population in 2018 for ASDR and 2019 for MSDR.

MISD technicians also participated in government‐led enforcement operations, which applied command‐and‐control policing (details in Appendix S1). These government‐led enforcement operations were conducted mainly outside the 2 studied PAs in large boats and involved different government agencies (e.g., IBAMA, environmental police, state environmental protection body). Data were recorded and managed by MISD on these operations in nearly the same period (2002–2012) covered by the VEA Program in the PAs. For each operation, data were recorded on the number of official agents involved, the operation time in days and hours, and the number of environmental crimes detected per operation. These data were used to compare the trends in the occurrence of crimes during VEA Program actions in the PAs versus the trends in number of crimes detected in government‐led enforcement operations outside PAs. The data we used were exclusively related to environmental crimes. The VEAs were always trained by MISD and supported by a government agency, either federal or state, depending on the year. Data were shared only with these agencies and with MISD, which was responsible for storing and managing the data. To determine how Indigenous peoples were involved in this practice, see Franco et al. (2021). We adhered to stringent ethical guidelines to protect all participants and individuals associated with the research. In accordance with the principles of the Declaration of Helsinki, we ensured that no personal data that could lead to the identification of individuals involved in environmental crimes were accessed or shared. This commitment to privacy and confidentiality was central to our methodology.

### Data analyses

For both the data from the VEA Program and government‐led enforcement operations, we used descriptive statistics to report the total number of patrols or operations carried out during the study period; average number of patrols or operations undertaken per year; total number of hours and average time spent in patrols or operations; average number of VEAs or official agents participating in each patrol or operation; total number of crimes; and average number of crimes per patrol or operation. For the VEA Program, we also calculated the percentage of patrols that were routine or were in response to an informant report.

To analyze the effectiveness of the VEA Program patrols in curbing environmental crimes, we used a generalized linear mixed model (GLMM) with the binomial family of distribution to assess the trend in the occurrence of crime detection during patrol outings over time in the 11‐year study period. Because VEA Program patrols during which more than one crime was detected were rare (only 9.7% of the total number of outings with infractions), we assigned 1 to patrols with crimes and 0 to those without crimes. Instead of pooling years, we transformed patrol dates into a continuous variable, with each patrol day treated as a sampling unit. By doing so, we aimed to capture the short‐term effects of patrol efforts and minimize potential temporal dependence that could arise from aggregating data over longer periods (e.g., years). Because the territorial unit covered by each group of patrollers remained constant over the years, we tested the effects of differential effort on crime detection by controlling for patrol effort. Specifically, we used the number of VEAs involved, patrolling time, and whether the patrol was routine or informant led as covariates in the model. This approach allowed us to address potential variations in patrol effort without resorting to a pooled catch‐per‐unit‐effort metric (Dancer et al., 2022).

To account for potential differences in effectiveness across territorial units, we included an interaction between time and territorial units in our model. To address temporal dependence and control for possible seasonality in crime occurrences, we used a mixed‐effects approach. In this model, territorial unit was a fixed factor, and year nested by month was a random factor. This nesting structure was crucial because patrols started at different times in each territorial unit, and it allowed us to account for both the staggered start dates of patrol efforts and seasonal patterns in the data. To ensure that effort exerted in patrols over time did not affect our results, we used GLMMs with the Gaussian family of distribution to test whether the time spent and number of VEAs involved in each outing changed over time, including territorial unit as a random effect.

For comparison, we used a generalized linear model (GLM) to assess the trends in crimes detected during the government‐led operations conducted outside the PAs. In contrast with the VEA outings, these operations usually lasted more than 1 day. Therefore, we transformed the initial and final dates of each operation into a continuous variable and took the mean of both values to use this as a reference for the period of each operation. For this model, we considered the count of crimes detected per operation as the response variable and used the negative binomial type I (NBI) family of distribution to assess the trend in the number of crimes detected during operations within the 10‐year period. We controlled for effort; we used the number of official agents involved and time spent in hours (transformed into natural log) as covariables. To ensure that effort exerted in government‐led enforcement operations did not affect our results, we tested whether the time spent and number of agents involved in each operation changed over time; we used a GLM with the gamma distribution for time and NBI distribution for number of agents. We used R 3.3.0 statistical software, the lubridate package to transform the dates into a continuous variable, and the gamlss package to conduct the GLMM and GLM. We considered *p* < 0.05 significant.

## RESULTS

### Characteristics of the voluntary patrolling and government‐led enforcement operations

The VEAs carried out 19,957 patrols in the PAs, an average of 1814 outings per year (SD 861). This totaled 149,864 patrol hours. Patrols lasted on average 7.51 h (SD 2.60) each. Around 95% (n = 18,935) of these were conducted for routine purposes, and 5% (1022) were informant led. On average, 3.4 VEAs (SD 1.9) participated in each outing. Crimes were recorded in 1188 (6%) patrols, totaling 1260 crimes, with a mean of 0.06 crimes per outing (SD 0.27). A total of 897 crimes were detected during routine patrols and 363 from informant‐led patrols. Of the 772 crimes for which we had data on items confiscated, most (78.24%, n = 604) were related to fishing infractions, 19.04% (147) to hunting, and 2.72% (21) to logging.

The government conducted 69 enforcement operations outside the PAs, an average of 6.9 operations per year. Operations involved on average 5.97 official agents (SD 1.95) and lasted 6.64 days (SD 2.99), or 159.30 h (SD 71.88). Crimes were detected in all operations (917 crimes, mean = 13.29 crimes per operation [SD 9.69]).

### Trends in environmental crimes

**FIGURE 2:**
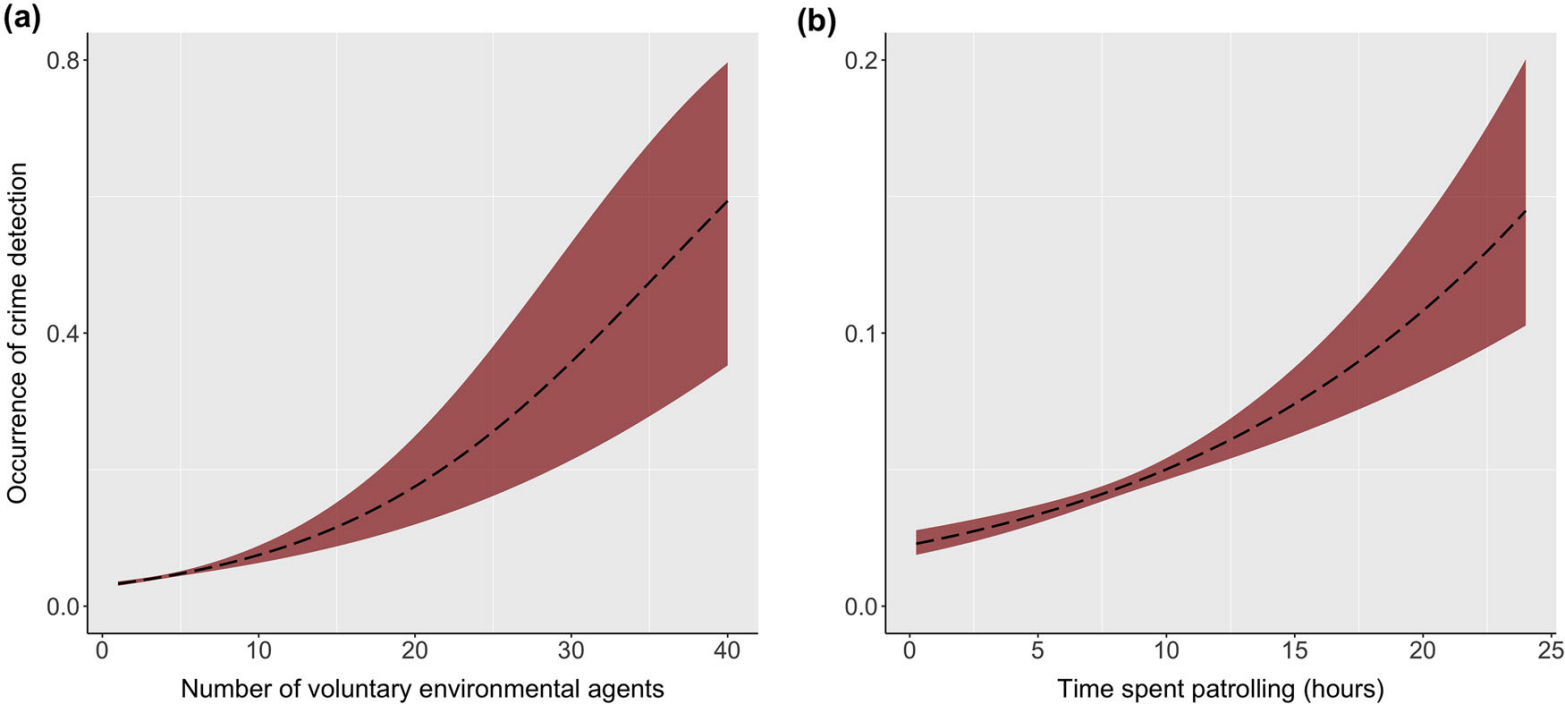
Occurrence of environmental crime detection in community‐based voluntary patrols in 2 reserves in the Brazilian Amazon relative to the (a) number of voluntary environmental agents involved and (b) time the agents spent patrolling.

**FIGURE 3:**
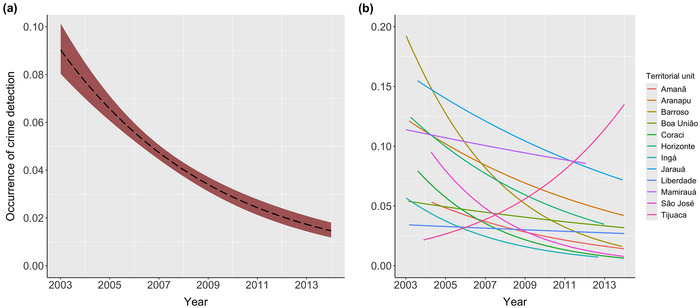
Trends in the occurrence of environmental crime detections during voluntary patrols from 2003 to 2013 for (a) all territorial units pooled and (b) each of the surveyed territorial units in 2 protected areas in the Brazilian Amazon.

For the VEA Program, the occurrence of crime detection increased as the number of VEAs involved increased (coefficient = 0.096 [SE 0.016], *t* = 6.22, *p* < 0.001) (Figure 2a) and as time spent increased (coefficient = 0.083 [0.012], *t* = 7.13, *p* < 0.001) (Figure 2b) in an outing. Detection of crimes was greater during outings that were informant led than during routine patrols (coefficient = −2.39 [0.08], *t* = −29.76, *p* < 0.001). There was a sharp decrease over time in the occurrence of crime detection events in patrols; overall reduction was around 80% across all units (from 9% to 2% of outings) during the study period, accounting for the staggered start of patrols in different territorial units (coefficient = −0.173 [0.08], *t* = −2.15, *p* < 0.05) (Figure 3a). Although the overall reduction remained significant across all units, the interaction highlighted that this trend was particularly strong in 11 of the 12 territorial units surveyed (Figure 3b) (statistical results for the full model in Appendix S2). Effort in terms of time spent (coefficient = 0.017 [0.026], *t* = 0.67, *p* = 0.50) and number of VEAs (coefficient = −0.032 [0.030], *t* = −1.06, *p* = 0.29) involved in patrols did not change over time (Appendix S3).

**FIGURE 4:**
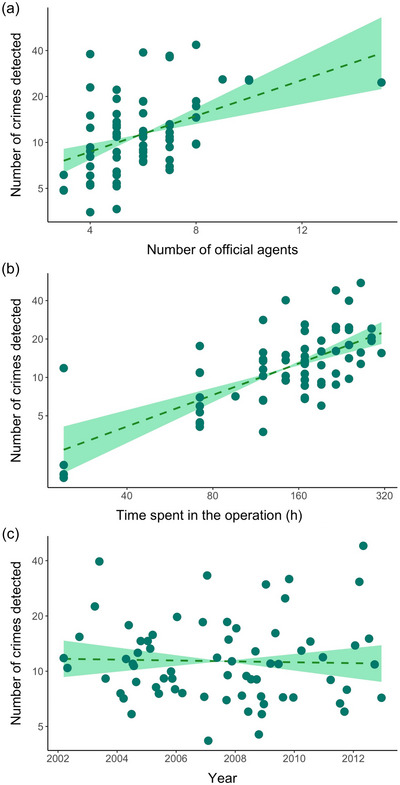
Number of environmental crimes detected during government‐led enforcement operations outside the 2 studied protected areas from 2002 to 2012 in the Brazilian Amazon relative to (a) the number of official agents involved, (b) time spent in the operation, and (c) year. Y‐axis is represented in natural log (ln) scale.

Similarly, for the government‐led enforcement operations outside PAs, the number of crimes detected per outing increased as the number of official agents involved increased (coefficient = 0.14 [0.03], *t* = 4.17, *p* < 0.0001) (Figure 4a) and the time spent (coefficient = 0.82 [0.12], *t* = 6.71, *p* < 0.0001) in the operation increased (Figure 4b). However, in contrast with the VEA Program in PAs, there was no clear reduction in the number of crimes detected over time (coefficient = −0.006 [0.005], *t* = −1.20, *p* = 0.237) (Figure 4c). Effort in terms of time spent (coefficient = 0.0004 [0.0018], *t* = 0.24, *p* = 0.81) and number of official agents (coefficient = 0.01 [0.02] *t* = 0.55, *p* = 0.582) involved in government‐led operations did not change over time (Appendix S4).

## DISCUSSION

There is an urgent need to find more cost‐effective ways of protecting tropical forests worldwide. One of the main concerns is how to curb crime that threatens wild species in areas that are important for the conservation of biodiversity and for the food security and livelihoods of people living in and around them. Protection against environmental crimes is particularly important where there is insufficient governmental enforcement. In these circumstances, the increased participation of local populations in community‐based actions and in the law enforcement process has been globally advocated (Bergseth et al., 2018; Danielsen et al., 2005; Franco et al., 2021; Sablayrolles et al., 2019). We brought together a unique set of long‐term patrol data that demonstrated that the VEA Program in 2 PAs in Brazil was effective in curbing environmental crimes. To reinforce the evidence of this community‐based voluntary patrolling system's success, we compared our results with the trends in crimes documented in government‐led enforcement operations outside these PAs. This comparison underscored the significant decrease in environmental crimes within the ambit of the community‐based system, starkly contrasting with the trends observed under government‐led enforcement efforts. This underscores the importance of advocating for community‐driven interventions, even beyond PAs, and emphasizing the necessity of incorporating local efforts into broader environmental protection initiatives.

Although it is not possible to rule out alternative explanations, such as problems in the system itself—where some patrollers may become more lenient or corrupt or law violators become more effective in avoiding detection—the fact that there was a reduction in crimes in 11 of the 12 analyzed independent territorial units provides strong support for the relationship between reduced occurrence of crimes detected during outings and a real decrease in the number of crimes occurring in these areas. Other limitations should be considered, including the reliability of crime reporting, potential biases in data collection, and the possibility that our methods may not produce the same results in different contexts or regions. Three other factors provide confidence in the VEA Program's effectiveness. First, patrolling and government‐led operation efforts were constant over time, suggesting that effectiveness was not due to increases in effort. Second, engagement in the VEA Program increased over time commensurate with community‐based management, and new areas were added to patrols (see Franco et al., 2021). This indicates a substantive level of engagement across time with perceived benefits leading to expansion into new areas. Third, from 2010 to 2015, the reduction of illegal activities in sustainable‐use PAs in the Brazilian Amazon—which allow people to live in them—has been greater than in strict PAs, and this pattern has been attributed to community surveillance (Kauano et al., 2017). The social and collective disorganization of territorial management, added to external factors, such as the presence of more illegal buyers in the communities, may explain the lack of reduction in environmental crimes in the only territorial unit that did not present a reduction in crimes. This condition caused this territorial unit to lose the necessary permits for managing forest products and arapaima fish. Developing strategies to address these challenges is crucial for enhancing governance and ensuring the effectiveness of patrolling efforts in this specific region.

The VEA Program exemplifies a comprehensive integration of local community involvement in PA law enforcement and aligns with key factors that facilitate effective conservation (Sharkey et al., 2024). The VEA Program works across multiple stages of the enforcement chain, including detection, threat removal, and reporting, embodying a hands‐on approach to conservation. The VEA Program participants have critical roles in decision‐making, especially concerning patrol routes and methods, which ensures that enforcement strategies are well suited to local conditions and needs. Support for VEAs comes from governmental and nongovernmental entities. These entities provide training, resources, and financial backing, which are critical for the program's operationalization. Likely, VEAs are driven by a blend of intrinsic values, such as heritage and livelihood protection, and extrinsic rewards, such as formal recognition and support from environmental bodies. This mix of motivations fosters deep‐rooted engagement and commitment, even when there are challenges. Moreover, their role is formalized through official recognition, yet they maintain the flexibility to act in informal capacities, which allows them to adapt to dynamic conservation challenges and enhance community compliance with environmental regulations.

Crimes detected based on alerts from local people represented a high proportion of the recorded infractions, demonstrating the importance of the engagement of local populations in supporting the legitimacy of VEA patrols. In a global assessment in marine PAs, inaction was the overall most common response of interviewed fishers who had witnessed an environmental violation in most of the studied areas (Bergseth et al., 2018). Social cohesion and trust are among the main factors that prompt the local population to support policies and report violations of environmental regulations (Bergseth et al., 2018). The communities in our study possess a strong sense of ownership over the territory, rooted not only in its historical use but also in the legitimacy acquired through PAs (Franco et al., 2021).

The effort spent in terms of the number of people and time patrolling also contributes to crime detection. The effectiveness of law enforcement often relies on the availability of financial resources, equipment, and training of agents (Hilborn et al., 2006; Struhsaker et al., 2005). This result highlights the need for governmental investments in equipment and support for the outings—such as fuel—and to expand training to allow more local people to participate in the VEA Program.

Making local community members key players in resource protection and including them in decision‐making processes is positively associated with stronger commitment to and support for voluntary patrolling worldwide (Bergseth et al., 2018; Gray e Kalpers, 2005; Kothari et al., 2013). Programs like the VEA Program not only promote participation in the surveillance process but also reinforce community‐based management carried out by local residents (Armitage, 2005). Although there is official training by the government and partner institutions, the VEA Program is unique because it is mostly based on voluntary work by the residents. Most rangers in other parts of the world, such as in African parks, are paid (e.g., Gray & Kalpers, 2005). Additional nonmonetary benefits, such as the protection of their natural resources for personal and collective use, possibly motivate community members to take part in the program. The VEA Program, therefore, creates conditions for the local communities to embody stewardship concepts and take responsibility for the sustainable use of resources, which ultimately leads to higher compliance with the local and official rules and agreements.

The VEA Program enabled the implementation of several additional strategies for the sustainable and economic use of biodiversity, including community‐based tourism, wildlife management, and improvement of local population well‐being (Franco, 2020; Franco et al., 2021). As a result of local empowerment, the population living around the PAs—who previously overexploited natural resources—later recognized the access rights of local communities and began respecting local rules (Amâncio, 2006). These local rules typically include community‐defined regulations that govern the sustainable use of natural resources, such as restrictions on hunting, fishing, and the harvesting of forest products. Traditional governance structures often involve community assemblies or councils, in which local leaders and elders play a critical role in decision‐making and enforcing rules. However, there is a concern that increased community responsibility could lead governments and official forces to reduce their support and resources, assuming communities can manage these on their own. It is vital to maintain a balance, where the local communities’ efforts are supported by consistent and effective government intervention that provides necessary resources, legal support, and broader management strategies.

Contemporary conservation challenges require coordinated and effective actions to protect biodiversity (Moon et al., 2021). Noncompliance with laws and local rules is a worldwide phenomenon in PAs (Atuo et al., 2020; Collins et al., 2021). Coordination between patrolling and management programs is crucial to boosting compliance (Ponta et al., 2021). For example, countries that implement fisheries management alongside strong surveillance capacity experience fewer illegal fisheries activities (Petrossian, 2015). The inclusion of local communities in the planning and conduction of surveillance actions can contribute to rule conformity and the protection of threatened species and landscapes (Atuo et al., 2020). Community‐based initiatives are an important complement to the government's command‐and‐control approach to environmental protection in developing countries (Keane et al., 2008; Rowcliffe et al., 2004). Considering compliance at appropriate levels is critical to guiding local and national natural resource management policies (Oyanedel et al., 2020). It is therefore important that natural resource protection and management agencies continue to develop programs that enhance compliance through voluntary surveillance and enforcement (Bergseth et al., 2018), increasing the effectiveness of local control systems.

**FIGURE 5:**
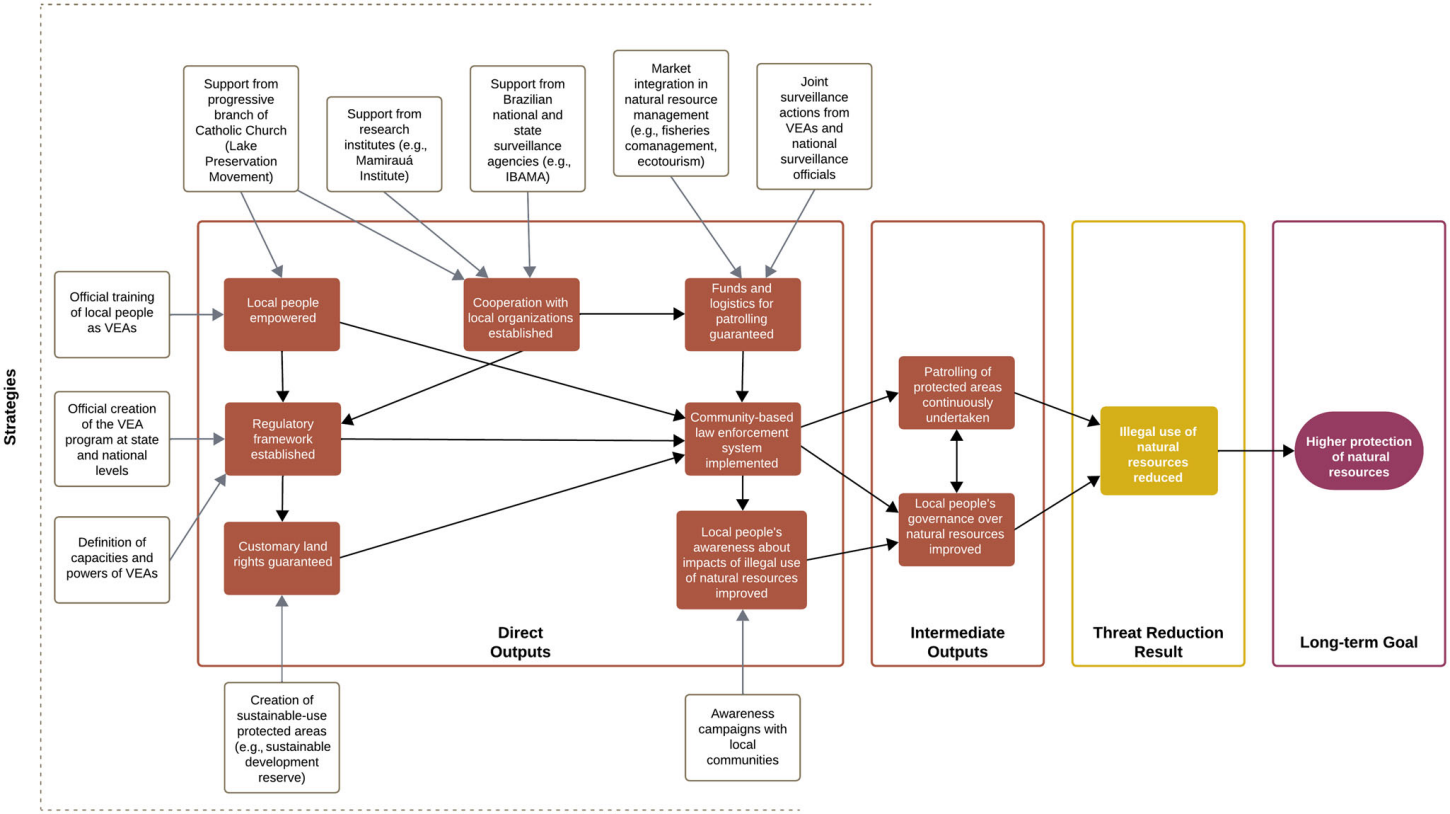
Theory of change illustrating the strategies (outer boxes), outputs, and goals involved in the establishment and effectiveness of the voluntary community‐patrolling program (VEA) in Amanã Sustainable Development Reserve and Mamirauá Sustainable Development Reserve (based on Franco et al. [2021]).

Although we observed a clear decline in environmental crimes in PAs, there were factors other than community patrolling that contributed. It is critical to recognize that although community‐led enforcement is an integral component, it operates within a broader context of collaborative management and benefit sharing, which together enhance the effectiveness of conservation strategies (Figure 5). These factors include natural resource management initiatives that involve the active responsibility of residents. These initiatives create socioeconomic and ecological benefits that affect local communities directly. Such benefits can significantly motivate community engagement in conservation efforts because residents experience firsthand the advantages of sustainable practices and natural resource protection. This holistic approach to management, exemplified by the long‐standing community involvement in these PAs, incorporates elements of citizen science and builds trust, fostering deeper participation in conservation. The combination of these elements, rather than an isolated community‐based enforcement, likely contributes to the observed compliance and support for conservation measures in these areas.

Comanagement models that involve collaboration among communities, state authorities, and supporting organizations provide an important foundation for the success of a community‐based voluntary patrolling system (see Franco et al., 2021). Key to this approach is fostering strong community engagement in designing and implementing conservation actions. This includes conducting environmental education, mobilizing community participation in resource management, and developing local leadership through training and capacity building. Partnerships with state bodies and institutions should be fostered to support the program's legal and operational framework and to provide resources, such as capacity building, funding, and technical assistance. Adapting the model to fit the specific cultural, ecological, and sociopolitical contexts of different regions is essential and allows for customized roles and responsibilities of community agents (Berkes, 2007; Ostrom, 2007). A flexible framework should be maintained that accommodates shifts in the intensity and nature of comanagement, partnerships, and cooperation over time. This adaptability ensures the program's sustainability and effectiveness across diverse settings. By integrating these elements—strong community involvement, supportive state partnerships, contextual adaptation, and a flexible management framework—conservationists can effectively extend community‐based environmental protection efforts worldwide to enhance local empowerment and global conservation outcomes.

The comprehensive patrolling efforts by VEAs culminated in a substantial body of data, reflecting significant dedication to monitoring and protecting PAs. Notably, an 80% reduction in the occurrence of crimes was recorded, demonstrating the effectiveness of the patrolling strategy. The strength of this strategy lies in its scalability, especially considering the vast and diverse expanse of the tropical region. Scaling this approach would require consideration of logistical support, the adaptability of rangers to diverse ecosystems, and the integration of local communities and technologies to improve efficiency and effectiveness. In essence, a balance among technological, human, and community resources will be fundamental to extending the reach of this strategy to other PAs around the globe.

## Supporting information



Supporting Information
