# Supplementary material for: Strengthening Amazon conservation through community‐based voluntary patrolling
Source: Conserv Biol. 2025 May 30;39(3):e70045. doi: 10.1111/cobi.70045 (PMC12124175; doi:10.1111/cobi.70045)
Supplement: Supplementary file 1 — Supporting Information [file COBI-39-e70045-s001.docx]

**Supplementary Material**

**Government-led enforcement operations**

Government-led enforcement operations primarily occur outside the two researched protected areas and are conducted using large boats. These operations involve collaboration between various government agencies such as the National Agency of Enforcement (IBAMA), environmental police, and state environmental protection bodies, as well as local institutions like MISD.

Between 2002 and 2012, a total of 69 government-led enforcement operations were carried out in this region. On average, approximately 6.3 missions were conducted each year. Each operation lasted, on average, about 6.5 days. On average, each operation covered around 500 kms. The majority of the operations were most prevalent in rivers, followed by lakes. Throughout these operations, an average of 14 infractions were recorded. Additionally, each operation involved an average of about 6 agents from various organizations, highlighting the collaborative efforts in environmental enforcement during this period.

**Table 1.** Details of the generalized linear mixed model for the assessment of the trend in the rate of observed crimes during patrol outings of the Voluntary Environmental Agents Program (VEA Program) over time within the 11-year period (2003-2013) within two reserves (Amanã Sustainable Development Reserve and Mamirauá Sustainable Development Reserve) in the Brazilian Amazon.

| Predictor variables | Estimate | Std. Error (SE) | t-value | p-value |
| --- | --- | --- | --- | --- |
| Intercept | 345.28 | 161.35 | 2.14 | 0.03 |
| Year | -0.17 | 0.08 | -2.15 | 0.03 |
| Number of environmental agents | 0.10 | 0.02 | 6.22 | < 0.0001 |
| Time spent patrolling (hours) | 0.08 | 0.01 | 7.13 | < 0.0001 |
| Reason (Routine) | -2.39 | 0.08 | -29.76 | < 0.0001 |
| Sector Aranapu | -127.91 | 176.62 | -0.72 | 0.47 |
| Sector Barroso | -13.63 | 177.96 | -0.08 | 0.94 |
| Sector Boa União | -286.33 | 179.62 | -1.59 | 0.11 |
| Sector Coraci | -78.20 | 203.23 | -0.39 | 0.70 |
| Sector Horizonte | -169.19 | 178.50 | -0.95 | 0.34 |
| Sector Ingá | -198.14 | 267.53 | -0.74 | 0.46 |
| Sector Jarauá | -139.73 | 187.87 | -0.74 | 0.46 |
| Sector Liberdade | -411.67 | 194.57 | -2.12 | 0.03 |
| Sector Mamirauá | -457.33 | 171.84 | -2.66 | 0.01 |
| Sector São José | 147.21 | 189.46 | 0.78 | 0.44 |
| Sector Tijuaca | -793.96 | 179.42 | -4.43 | < 0.0001 |
| Year:Sector Aranapu | 0.06 | 0.09 | 0.73 | 0.47 |
| Year:Sector Barroso | 0.01 | 0.09 | 0.08 | 0.94 |
| Year:Sector Boa União | 0.14 | 0.09 | 1.60 | 0.11 |
| Year:Sector Coraci | 0.04 | 0.10 | 0.38 | 0.70 |
| Year:Sector Horizonte | 0.08 | 0.09 | 0.95 | 0.34 |
| Year:Sector Ingá | 0.10 | 0.13 | 0.74 | 0.46 |
| Year:Sector Jarauá | 0.07 | 0.09 | 0.75 | 0.45 |
| Year:Sector Liberdade | 0.20 | 0.10 | 2.11 | 0.03 |
| Year:Sector Mamirauá | 0.23 | 0.09 | 2.67 | 0.01 |
| Year:Sector São José | -0.07 | 0.09 | -0.78 | 0.44 |
| Year:Sector Tijuaca | 0.40 | 0.09 | 4.43 | < 0.0001 |

**Table 2.** Details of the generalized linear model for the assessment of the trend in the number of observed crimes during outings of government-led enforcement operations over time within the 11-year (2002-2012) period outside PAs in the Brazilian Amazon.

| Predictor variables | Estimate | Std. Error (SE) | t-value | p-value |
| --- | --- | --- | --- | --- |
| Intercept | 8.62 | 9.23 | 0.93 | 0.354 |
| Time spent patrolling (log10 hours) | 0.82 | 0.12 | 6.71 | < 0.0001 |
| Year | -0.006 | 0.005 | -1.20 | 0.237 |
| Number of agents | 0.14 | 0.03 | 4.17 | < 0.0001 |


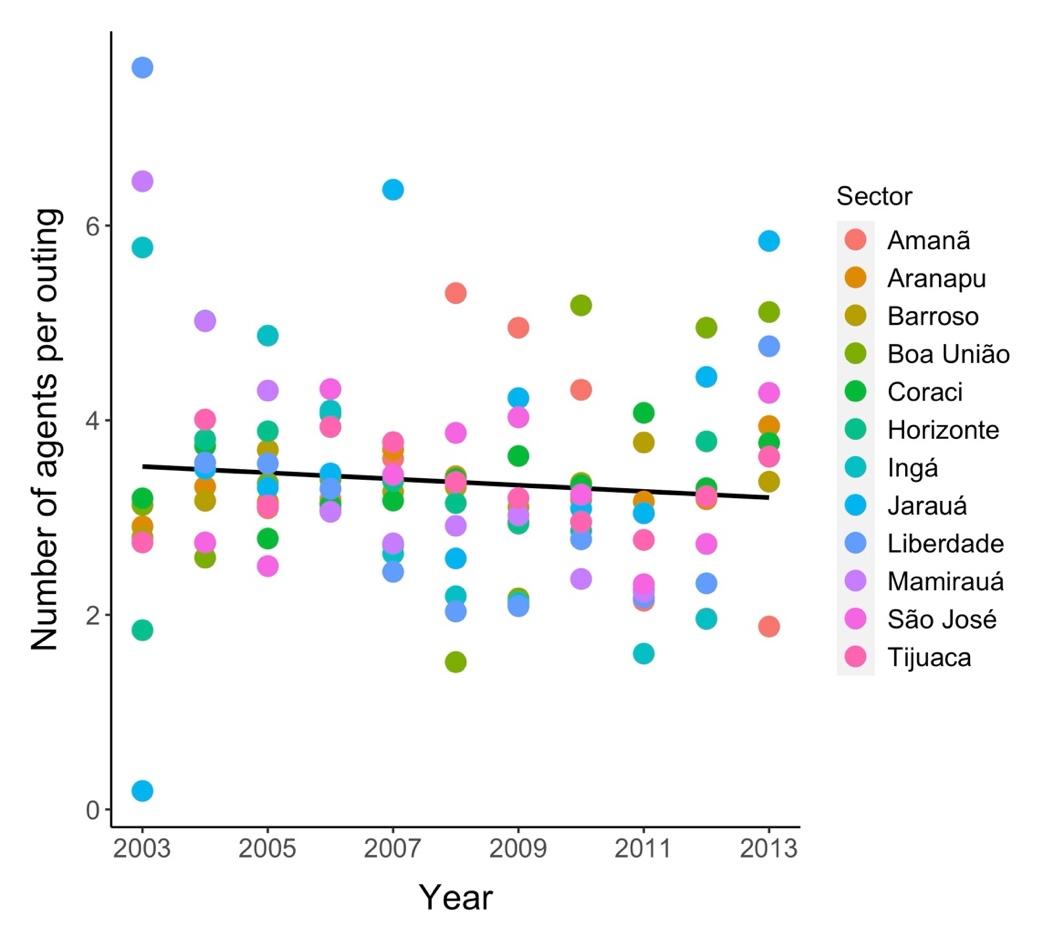


**Figure 1.** Relationship between time in years and effort in terms of number of voluntary environmental agents per patrol outing in the 12 sectors (territorial units) of the two protected areas analyzed on the effectiveness of community-based patrolling in curbing environmental crimes in the Brazilian Amazon.


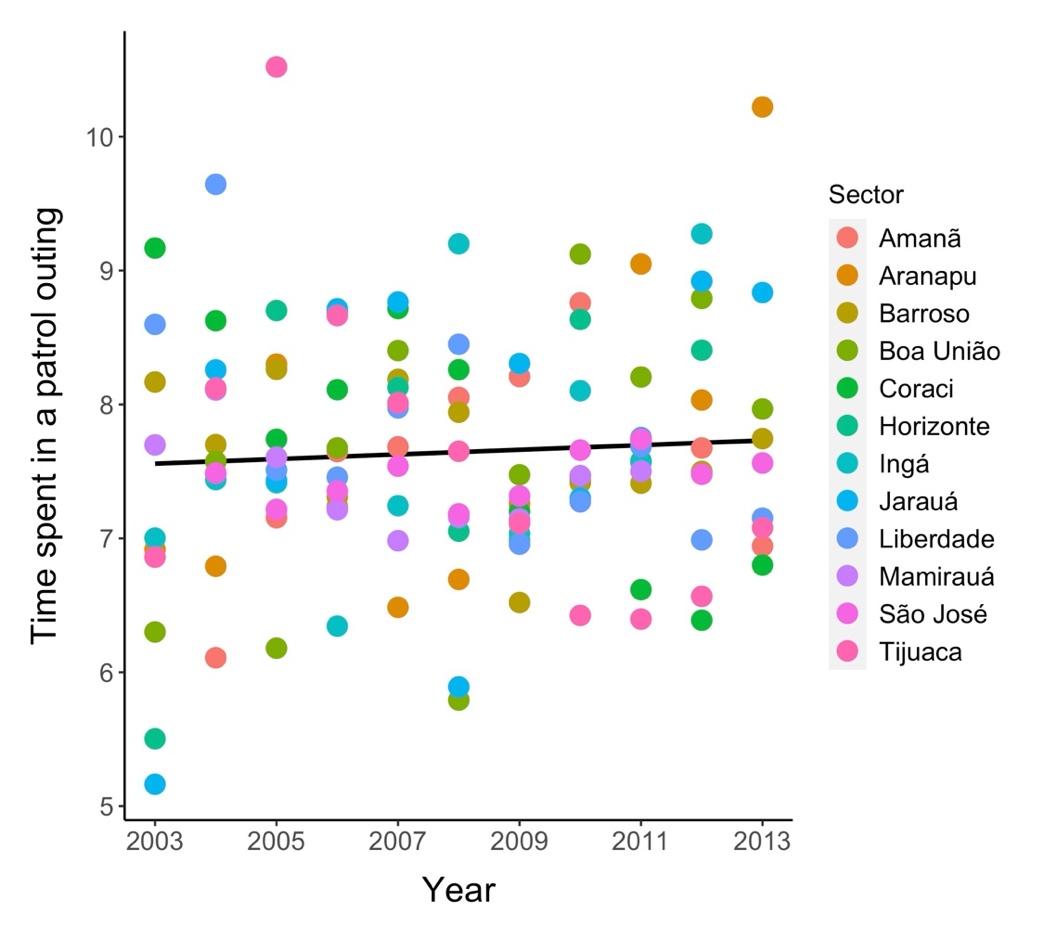


**Figure 2.** Relationship between time in years and effort in terms of time spent in patrol outings (in hours) in the 12 sectors (territorial units) of the two protected areas analyzed on the effectiveness of community-based patrolling in curbing environmental crimes in the Brazilian Amazon.

**
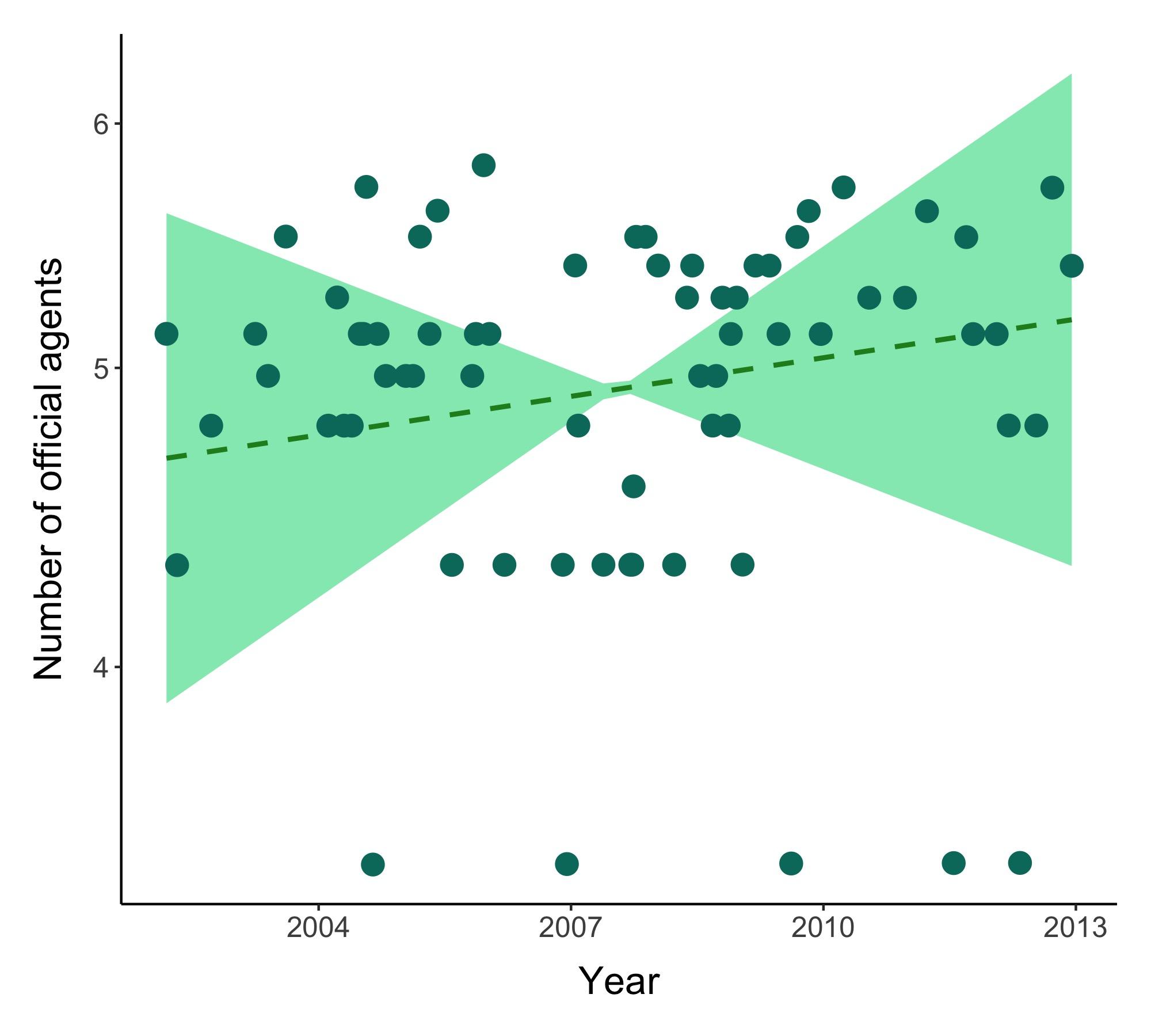
**

**Figure 3.** Relationship between time in years and effort in terms of number of official agents per government-led operation outside the two protected areas analyzed.


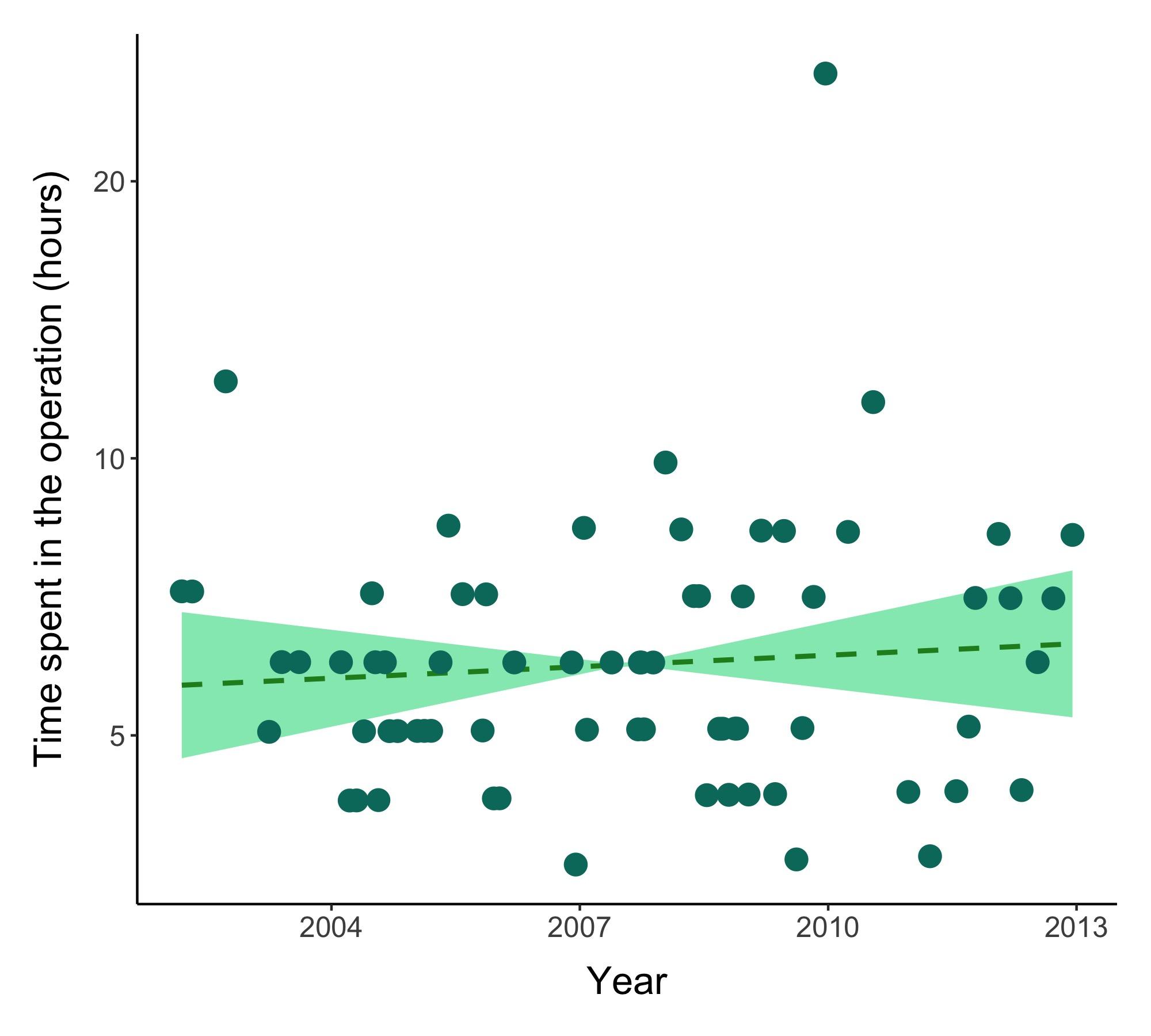


**Figure 4.** Relationship between time in years and effort in terms of time spent per government-led operation (in hours) outside the two protected areas analyzed.

**
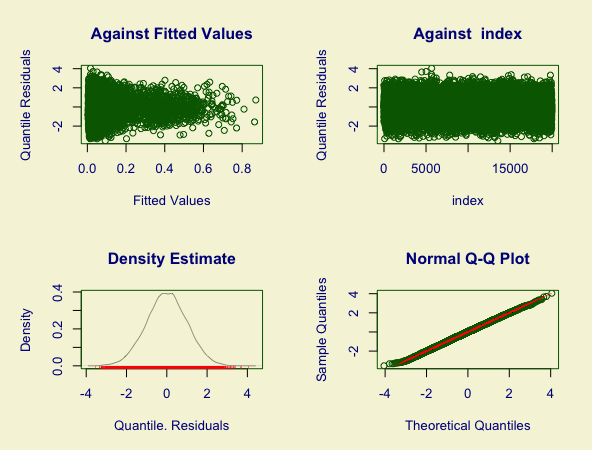
**

**Figure 5:** Diagnostic plots for the generalized linear mixed model (GLMM) using the Binomial family of distribution for the Voluntary Environmental Agents (VEA) program. The top-left plot shows normalized residuals against fitted values, helping assess the distribution of residuals across the range of predicted values. The top-right plot displays normalized residuals against the index (observation number), evaluating the independence of residuals over the sequence of observations. The bottom-left plot presents a density estimate of normalized residuals to assess their distribution, while the bottom-right plot is a Q-Q plot comparing the normalized residuals against theoretical quantiles.
